# Preliminary Investigation into a Potential Role for Myostatin and Its Receptor (ActRIIB) in Lean and Obese Horses and Ponies

**DOI:** 10.1371/journal.pone.0112621

**Published:** 2014-11-12

**Authors:** Philippa K. Morrison, Chen Bing, Patricia A. Harris, Charlotte A. Maltin, Dai Grove-White, Caroline McG. Argo

**Affiliations:** 1 University of Liverpool, Department of Obesity and Endocrinology, Faculty of Health and Life Sciences, Leahurst Campus, Neston, Wirral, United Kingdom; 2 Equine Studies Group, WALTHAM Centre for Pet Nutrition, Waltham-on-the-Wolds, Melton Mowbray, Leicestershire, United Kingdom; Faculty of Biology, Spain

## Abstract

Obesity is a widespread problem across the leisure population of horses and ponies in industrialised nations. Skeletal muscle is a major contributor to whole body resting energy requirements and communicates with other tissues through the secretion of myokines into the circulation. Myostatin, a myokine and negative regulator of skeletal muscle mass, has been implicated in obesity development in other species. This study evaluated gene and protein expression of myostatin and its receptor, ActRIIB in adipose tissues and skeletal muscles and serum myostatin concentrations in six lean and six obese animals to explore putative associations between these factors and obesity in horses and ponies. Myostatin mRNA expression was increased while ActRIIB mRNA was decreased in skeletal muscles of obese animals but these differences were absent at the protein level. Myostatin mRNA was increased in crest fat of obese animals but neither myostatin nor ActRIIB proteins were detected in this tissue. Mean circulating myostatin concentrations were significantly higher in obese than in lean groups; 4.98 ng/ml (±2.71) and 9.00 ng/ml (±2.04) for the lean and obese groups, respectively. In addition, there was a significant positive association between these levels and myostatin gene expression in skeletal muscles (average R^2^ = 0.58; p<0.05). Together, these results provide further basis for the speculation that myostatin and its receptor may play a role in obesity in horses and ponies.

## Introduction

Epidemiological studies continue to report a high prevalence of obesity amongst the leisure population of horses and ponies in the UK [1], [2]. The well-documented negative impacts of obesity on health and performance have led to obesity being considered one of the major welfare issues in horses and ponies facing industrialised nations today [3]. Obese animals are at an increased risk of developing insulin dysregulation and the severely painful and often life-threatening condition, laminitis, although the precise mechanisms linking these conditions are not yet fully understood.

The organ systems involved in energy homeostasis work in synergy to achieve the maintenance of whole body energy balance. As the largest metabolically active tissue in the body (comprising around 40% body mass, [4], [5]), skeletal muscle is a key determinant of resting energy expenditure and therefore plays a vital role in maintaining energy balance. Communication with other organs, including adipose tissue, is achieved through the secretion of molecular messengers into the circulation, termed myokines. Myostatin, a member of the transforming growth factor β (TGFβ) family of secreted growth factors, is one such myokine. The initial studies showed that mice lacking the myostatin gene were extremely hypermuscular and had minimal body fat when compared to their wild-type counterparts [6]. To date, myostatin has been widely characterised as a potent negative regulator of skeletal muscle mass [7]–[9] and methods to inhibit myostatin function as a potential therapeutic treatment for increasing muscle mass in diseases such as muscular dystrophy and cancer cachexia have been explored [10], [11].

Myostatin is synthesised as an inactive precursor protein which subsequently undergoes two cleavages to produce the mature, active form of the protein. Mature myostatin is bound noncovalently to its propeptide and circulates in serum as an inactive complex [12]. Active, mature myostatin binds selectively to the activin type II receptor kinase, ActRIIB. Studies in rodents and humans generally report that myostatin expression levels are highest in skeletal muscle, although it has also been identified in adipose tissue [6]. Previous work from this laboratory supports these findings and extends them to the horse. These data confirmed that myostatin gene and precursor protein expression is greatest in skeletal muscles and that in the horse, although low levels of expression were detected in adipose tissue at the gene level, myostatin precursor protein was absent [13].

Work in murine models and humans has identified that myostatin may have an important role in obesity development. Myostatin knock-out (KO) mice offered high-fat diets are resistant to gains in body fat [14], [15], and although this effect may be secondary to the increases in lean body mass, myostatin had direct effects on adipocyte differentiation [16], [17]. Furthermore, blocking myostatin increased the functional capacity of brown adipose tissue (BAT) [18] and may even drive the browning of white adipose tissue through the up-regulation of BAT-specific genes [19]. Myostatin gene expression was positively associated with obesity in both mouse [20] and human studies [21], whilst blocking myostatin function in mature mice elicited positive effects on glucose and insulin dynamics [22]. In comparison to human and rodent studies, there are fewer studies of myostatin in horses and ponies, and the extant reports generally focus on the identification of a number of single nucleotide polymorphisms (SNP’s) in the myostatin gene. SNPs have been associated with different attributes including breeds of different morphological type [23], optimal race distance in Thoroughbred horses [24] and skeletal muscle fibre type proportions in Quarter horses [25].

To date, no work has been conducted to characterize the expression myostatin and its receptor against the setting of obesity in the horse or pony. The current study was designed to explore possible differences in myostatin and ActRIIB expression between lean and obese animals by quantifying myostatin and ActRIIB gene and protein expression in skeletal muscle and adipose tissue, and measuring serum myostatin concentrations.

## Methods

### Animals and tissue collection

Although animal procedures did not constitute an experiment as defined under the Animals (Scientific Procedures) Act 1986, all work was approved by the University of Liverpool’s Veterinary Research Ethics Committee. Tissues from six lean (body condition score (BCS)/9 = 3.07±0.50, where 1 = emaciated and 9 = obese [26]) and six obese (BCS/9 = 7.7±0.46) mature, mixed breed horses and ponies were obtained *post-mortem*. All animals were in good general health and were euthanased for reasons unrelated to this study (Table 1). The horses were slaughtered in a commercial abattoir (LJ Potters, Taunton, Somerset) in accordance with EU legislations EC 852/2004, 853/2004 and 854/2004 on several dates between March 2013 and January 2014. *Ante-mortem* data collection included BCS (/9, [26]), breed type, gender, estimated withers height and age. For assessment of BCS, six areas of the body (neck, withers, loin, tailhead, ribs and shoulder) are assigned a number from 1 (emaciated) to 9 (obese) based on detailed descriptors. The average of these six numbers is calculated and this number equates to the final BCS score for the animal [26].

**Table 1:** Phenotypic descriptors for the animals used in this study.

	Horse ID	Gender	Age (years)	BCS (/9)	Breed type
Lean	1	Gelding	8	3	Welsh Pony
	2	Mare	5	3.8	Welsh Pony
	3	Gelding	15	2.5	Sport horse
	4	Gelding	6	3	Sport horse
	5	Mare	10	3.5	Sport horse
	6	Gelding	4	2.6	Sport horse
Obese	7	Mare	6	7	Cob horse
	8	Mare	13	8	Cob pony
	9	Mare	5	7.3	Sport horse
	10	Gelding	15	8.2	Cob pony
	11	Gelding	7	7.9	Cob pony
	12	Mare	15	7.8	Cob horse

Body condition score (BCS), age and gender are indicated. Breed types are as denoted in animal passports and/or confirmed by visual inspection. The term ‘cob’ denotes a small horse (>148 cm withers height) or large pony (<148 cm) with characteristically ‘heavy’ skeletal conformation. Sport horse is used to indicate animals of lighter type with Thoroughbred or Warmblood influenced conformation.

To evaluate gene and protein expression of myostatin and ActRIIB, a total of five anatomically-discrete adipose depots and four functionally distinct skeletal muscles were sampled. Strict anatomical descriptors were used to ensure that tissue samples were collected from the same site in each animal (Table 2). Tissue samples were obtained as rapidly as possible *post-mortem* (adipose tissues within 30 minutes; skeletal muscles within 1 hour) using sterile equipment, as recommended previously [13]. All samples were minced with scissors and snap frozen in liquid nitrogen before being stored at −80°C pending RNA and protein extraction. For the measurement of myostatin protein in serum, blood samples (∼10 ml) were collected into plain tubes (BD Vacutainer) at exanguination and allowed to clot before centrifuging at 2000 g for 10 minutes at 4°C. Serum was collected and stored at −20°C pending myostatin protein measurement by ELISA.

**Table 2:** Specific anatomical descriptors used to locate the tissue collection points for the 5 regionally discrete adipose tissue depots and 4 skeletal muscles sampled from horses used in the current study.

Tissue		Anatomical descriptors for sample sites
Adipose tissues	Ventro-abdominal	∼3 cm^3^, collected from the left split-carcass midline at a point equidistant between xiphisternum and pubis.
	Epicardial	∼2 cm^3^ from the coronary groove and overlying the left coronary artery
	Omental	Variable area of omentum, sufficient to harvest ∼2 cm^3^ of adipose tissue, from a region adjoining the greater curvature of the stomach and bearing visible adipose.
	Crest	∼3 cm^3^ from the left split-carcass at the deepest part of the crest, midway between wither and poll extremities.
	Tailhead	∼2 cm3 from the subcutaneous adipose tissue overlying the gluteal muscles of the left carcass.
Skeletal muscles	*Rectus abdominis,*	∼3 cm^3^, collected from the left split-carcass midline at a point equidistant between xiphisternum and pubis.
	*Longus colli*,	∼3 cm^3^, from its severed cranial extremity in the left split-carcass.
	*Pectoralis transversus*	∼3 cm^3^, collected from the exposed midline section of the muscle at a point just ∼10 cm caudal to the thoracic inlet.
	*Pectoralis profoundus*	∼3 cm^3^, collected from the exposed midline section of the muscle, immediately deep to the collection site for *Pectoralis transversus.*

Approximate target sample sizes are given. Where relevant, tissues were collected from the left side following carcass-splitting.

### RNA extraction

Total RNA was extracted from all frozen tissue samples using TRIzol reagent (Invitrogen, Paisley, UK), in accordance with the manufacturers protocol. RNA concentration and purity was quantified spectrophotometrically (Eppendorf Biophotometer, Hamburg, Germany) and all optical density A260/280 ratios were within acceptable ranges (1.7–2.0). Reverse transcription (RT) was carried out in a 10 µl final reaction volume containing 0.5 µg RNA using an iScript cDNA synthesis kit (Bio-Rad Hemel Hempstead, UK). The resulting cDNA was diluted at 1∶4 and used as a template for real-time PCR analysis.

### Quantitative Real-Time PCR

The expression of myostatin, ActRIIB and four housekeeping genes previously used in other studies in horses and ponies [27], [28] (GAPDH, Beta-actin, HPRT1 and RPL32) was determined in all tissues from the twelve animals. GeNorm software (GenEx, Germany) was used to assess the two ‘most stably’ expressed genes to be used for normalisation. Gene expression was determined by quantitative real-time PCR performed in duplicate using the Stratagene Mx3005P detection system (Agilent Technologies, California USA). Primer sequences for all four housekeeping genes were obtained from previously published data (HPRT1 and RPL32, GAPDH [28], and beta-actin [27]) and 100% homology was confirmed by performing a basic local alignment search tool (BLAST). Primer and Taqman probe sequences for myostatin and ActRIIB, were designed using Beacon Designer (Premier Biosoft, USA). All primers were designed to be exon-spanning. All primer/probe sets were purchased from Eurogentec (Belgium) (Table 3). Serial dilutions of pooled cDNA were used to calculate Taqman primer efficiencies. The PCR cycling conditions (using Taqman probe and primers) for myostatin and ActRIIB were as follows: 10 minutes at 95°C, followed by 40 cycles of 30 seconds at 95°C, 1 minute at 55°C and 1 minute at 72°C. Cycling conditions for housekeeping genes (using SYBR green method) were as follows: 10 minutes at 95°C followed by 40 cycles of 15 seconds at 95°C and 30 seconds at 60°C and ending with, 1 minute at 95°C, 30 seconds at 55°C and 30 seconds at 95°C.

**Table 3:** Nucleotide sequences of primers and probes used in the current study.

Gene	Primer	Sequence	Amplification efficiency
Beta-actin	Forward	GGACCTGACGGACTACCTC	97%
	Reverse	CACGCACGATTTCCCTCTC	
HPRT1	Forward	GGCAAAACAATGCAAACCTT	94.5%
	Reverse	CAAGGGCATATCCTACGACAA	
GAPDH	Forward	CAGAACATCATCCCTGCTTC	95%
	Reverse	ATGCCTGCTTCACCACCTTC	
RPL32	Forward	AGCCATCTACTCGGCGTCA	94%
	Reverse	TCCAATGCCTCTGGGTTTC	
Myostatin	Forward	GCAGTGATGGCTCTTTGGAAG	97.9%
	Reverse	GCATTAGAAGATCAGACTCTGTAGG	
	Probe	ACCACGCGACGACGGAAACAATCAT	
ActRIIB	Forward	GCCTCGCTGTTCGGTTTGAG	92.9%
	Reverse	GGCTCCCTCAAGCACCTCAG	
	Probe	ACCGCCGTGTGCCCACCTGC	

Relative gene expression was calculated using the comparative Ct method (2^−ΔCt^) [29]. All gene expression data were normalised to 2 internal housekeeping genes.

### Protein Extraction and Western Blotting

Soluble protein was extracted from frozen tissues by homogenising around 100 mg of tissue in a SHE buffer (250 mM sucrose, 1 mM HEPES, 0.2 mM EDTA) containing both phosphatase and protease inhibitor cocktails (both Sigma, Poole, Dorset, UK). Samples were centrifuged and the soluble fraction was used for determining protein concentration by the bicinchoninic acid (BCA) method [30].

Thirty micrograms of protein extract were separated on 10% SDS-polyacrylamide gels under reducing conditions and proteins were transferred onto nitrocellulose membranes (Hybond-C Extra, Amersham Bioscience, Buckinghamshire, UK) by electroblotting (Turbo transfer, BioRad). Membranes were stained in Ponceau S reversible stain to verify the success of protein transfer and then blocked for 1 hour in 5% BSA in Tris-buffered saline containing 0.1% Tween 20 (TBST). Commercially available primary antibodies (listed below) were used and were selected on the basis that they were listed as having ‘equine cross-reactivity’. They were added at the following concentrations: myostatin precursor (MSTN), 1∶2500 [ab98337 Abcam, Cambridge, UK], myostatin receptor (ACTRIIB), 1∶2000 [sc-25453 Santa Cruz, Dallas, Texas, USA] and the serine/threonine Akt (AKT), 1∶3000 [#9272 Cell Signalling, Danvers, MA, USA]) in blocking buffer and incubated overnight at 4°C. The myostatin antibody detected the precursor form of the protein (∼43 kDa). The membranes were washed and then incubated for 1 hour with a secondary antibody (Cell Signalling) at appropriate concentrations. Signals were detected by chemiluminescence using a SuperSignal West Pico Chemiluminescent Substrate (Pierce, Rockford, IL, US) and visualised and quantified on a Molecular Imager ChemiDoc XRS+ System (Bio-Rad). The results were normalised to the value of AKT. To ensure the reliability of data, western blots for myostatin and ActRIIB proteins in skeletal muscles were repeated three times and average densitometric values were calculated.

### Myostatin ELISA

Mature myostatin protein concentration was measured using a commercially available ELISA kit (R&D Systems, Catalogue number: DGDF80) which has been validated for use on ‘equine serum and plasma samples’ by R&D systems (www.rndsystems.com/pdf/DGDF80.pdf) and employs the quantitative sandwich enzyme immunoassay technique to measure mature myostatin concentration. Prior to running the plate, samples were subjected to acid activation and neutralisation to remove the pro-peptide from myostatin. Samples were run in duplicate and the ELISA was run according to the manufacturer’s protocol. Myostatin concentration (ng/ml) was calculated from a standard curve.

### Statistical Analysis

Statistical analyses were performed using STATA version 12.1. Non-parametric, analytical methods were employed to assess gene and protein expression data. The Kruskal Wallis test was used to assess differences in gene and protein expression, along with differences in circulating myostatin concentrations between lean and obese animals. Associations between circulating myostatin and myostatin plus ActRIIB gene and protein expression were analysed using linear regression. Significance was set at p<0.05.

## Results

### Animals

The animals used in this study were slaughtered in a commercial abattoir for non-research purposes. The BCS in the population fell within the commercial range and lean and obese BCS categories were selected to give clear differences in body fat content [31].

### Myostatin and ActRIIB gene expression

**Figure 1:**
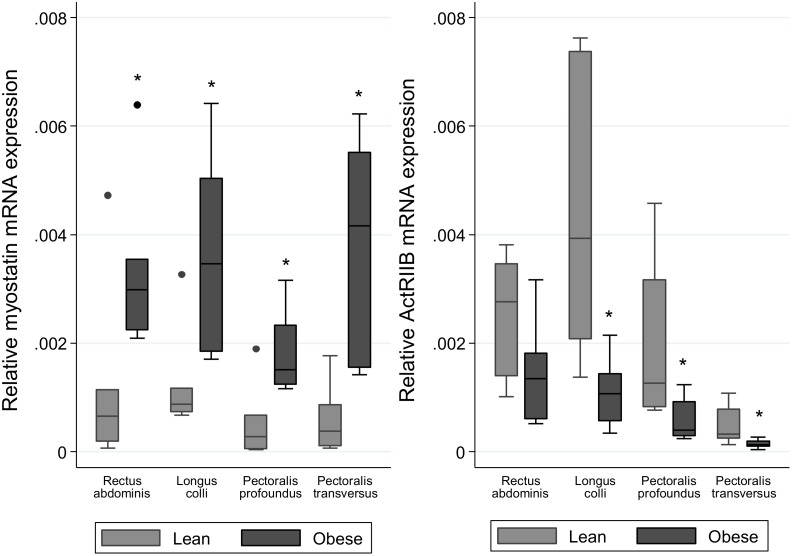
Gene expression of Myostatin and ActRIIB in skeletal muscles of lean and obese horses and ponies. Gene expression was analysed by real-time PCR and the geometric mean of the two most stable housekeeping genes as determined by GeNorm (GAPDH and beta-actin) was used for normalisation. Relative transcript abundance is shown for Myostatin (A) and ActRIIB (B). *denotes where values differ significantly (p<0.05) from lean group. n = 6/group.

**Figure 2:**
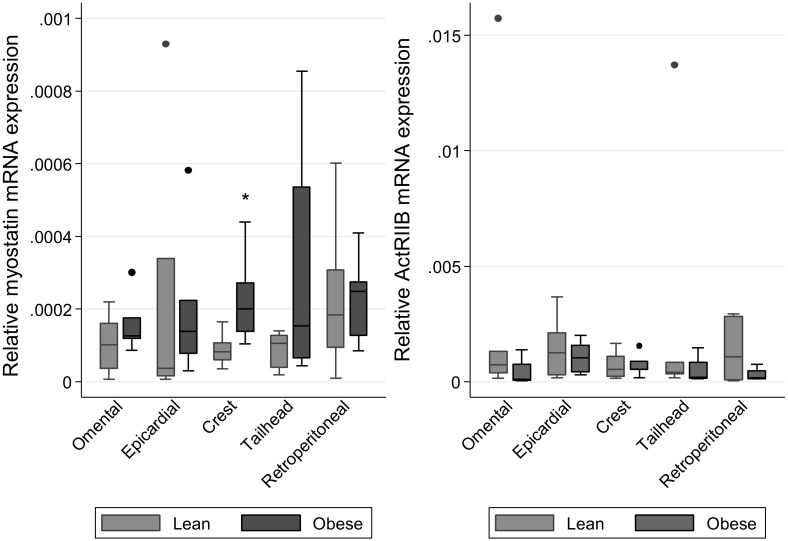
Gene expression of Myostatin and ActRIIB in adipose tissues of lean and obese horses and ponies. Gene expression was analysed by real-time PCR and the geometric mean of the two most stable housekeeping genes as determined by GeNorm (RPL32 and beta-actin) was used for normalisation. Relative transcript abundance is shown for Myostatin (A) and ActRIIB (B). *denotes where values differ significantly (p<0.05) from lean group. n = 6/group.

Myostatin gene expression across all skeletal muscles studied was significantly greater in the obese animals compared to the lean animals (p<0.05) (Figure 1a). In contrast, ActRIIB gene expression was significantly lower in obese animals in three out of the four skeletal muscles studied (p<0.05) (Figure 1b). While myostatin gene expression was considerably lower in adipose tissues in comparison to skeletal muscles, expression was significantly greater in the crest fat of obese animals compared with lean animals (p<0.05) (Figure 2a). No difference was observed between lean and obese animals for ActRIIB gene expression in adipose tissues (Figure 2b).

### Myostatin and ActRIIB protein expression

**Figure 3:**
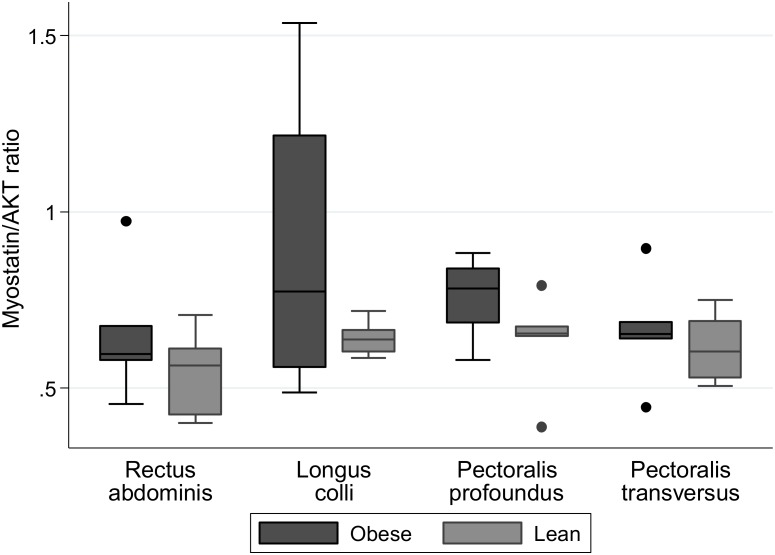
Protein expression of Myostatin precursor protein in skeletal muscles of lean and obese horses and ponies. Protein expression of myostatin precursor protein (∼45 kDa) was assessed by Western blot with total AKT used as a loading control. Mean densitometric signals were calculated from three independently repeated Western blots. n = 6/group.

**Figure 4:**
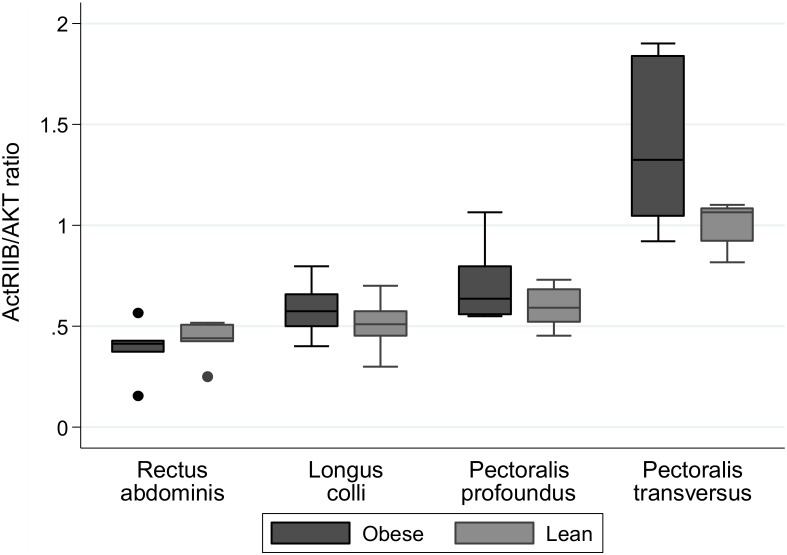
Protein expression of ActRIIB protein in skeletal muscles of lean and obese horses and ponies. Protein expression of ActRIIB protein (∼50 kDa) was assessed by Western blot with total AKT used as a loading control. Mean densitometric signals were calculated from three independantly repeated Western blots. n = 6/group.

**Figure 5:**
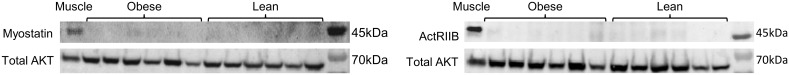
Protein expression of Myostatin precursor and ActRIIB proteins in crest fat of lean and obese horses and ponies. Protein expression of myostatin precursor protein and ActRIIB was assessed by Western blot with total AKT used as a loading control. Blots are shown for crest fat expression across all animals with a skeletal muscle sample used as positive control. n = 6/group.

Myostatin precursor protein expression was quantified across the four skeletal muscles by western blotting in three separate experiments. Although the average densitometric data showed no significant differences between lean and obese animals for any skeletal muscle studied (*Pectoralis transversus*, p = 0.75; *Longus colli*, p = 0.42; *Rectus abdominis*, p = 0.26; *Pectoralis profoundus*, p = 0.08), obese animals tended to have greater myostatin protein expression compared with lean animals (Figure 3). Similarly ActRIIB protein expression was quantified across the four skeletal muscles by western blotting in three separate experiments. No significant differences in protein expression between lean and obese animals were observed in the skeletal muscles studied (Figure 4). Due to the differences observed at the gene level in crest fat for myostatin, we sought to identify whether these differences were translated into differences at the protein level for both myostatin and ActRIIB. However, Figure 5 clearly demonstrates there was no protein detected in either the lean or obese animals for myostatin or ActRIIB.

### Circulating myostatin concentration

**Figure 6:**
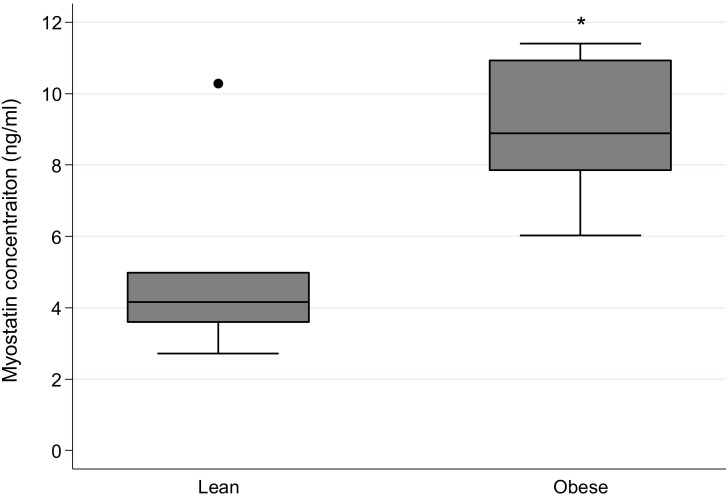
Circulating concentrations of myostatin protein in lean and obese horses and ponies. Circulating concentrations of mature myostatin protein were measured in blood serum using a commercially available ELISA kit. *denotes where values differ significantly (p<0.05) from lean group. n = 6/group.

Circulating, mature myostatin protein was detected in serum samples from all animals studied. Overall, mean serum myostatin concentration was 6.99 ng/ml (±3.10); the range was 2.72 ng/ml to 11.40 ng/ml. The mean values for the lean and obese groups were 4.98 ng/ml (±2.71) and 9.00 ng/ml (±2.04), respectively. Kruskal Wallis test revealed significant differences between lean and obese animals (p<0.05) (Figure 6). Univariate analysis revealed positive associations between serum myostatin concentrations and myostatin mRNA expression in skeletal muscle for all muscles studied, irrespective of whether muscles were considered independently or collectively (average R^2^ = 0.58, p<0.05) (Table 4). Associations between myostatin serum concentrations and the magnitude of muscle myostatin protein expression were weaker than those recorded for gene expression (average R^2^ = 0.28). Only myostatin protein expression in *Pectoralis profoundus* had a significant association with serum myostatin concentration (R^2^ = 0.50, p = 0.01) (Table 4). No associations were identified between myostatin serum concentration and ActRIIB gene or protein expression (Table 4).

**Table 4:** Univariate regression analysis results for myostatin ELISA data.

Variable		Coefficient	Adjusted R^2^	95% CI	P value
Myostatin gene expression	*Rectus abdominis*	1287.62	0.65	622.89 to 1952.35	0.002
	*Longus colli*	1179.68	0.54	409.69 to 1949.68	0.007
	*Pectoralis profoundus*	2811.07	0.78	1761.03 to 3861.11	<0.001
	*Pectoralis transversus*	836.77	0.36	57.41 to 1616.13	0.04
ActRIIB gene expression	*Rectus abdominis*	−201.53	0.01	−2083.14 to 1680.08	0.82
	*Longus colli*	−583.66	0.23	−1336.72 to 169.40	0.12
	*Pectoralis profoundus*	−1332.49	0.32	−2710.86 to 45.89	0.06
	*Pectoralis transversus*	−5812.13	0.33	−12115.38 to 491.11	0.07
Myostatin protein expression	*Rectus abdominis*	7.28	0.13	−6.17 to 20.74	0.26
	*Longus colli*	5.36	0.26	−0.95 to 11.67	0.09
	*Pectoralis profoundus*	16.40	0.50	4.81 to 27.99	0.01
	*Pectoralis transversus*	12.58	0.24	−3.39 to 28.54	0.11
ActRIIB protein expression	*Rectus abdominis*	−8.91	0.15	−23.98 to 6.15	0.22
	*Longus colli*	1.43	0.004	−13.84 to 16.71	0.84
	*Pectoralis profoundus*	10.35	0.25	−2.37 to 23.08	0.10
	*Pectoralis transversus*	6.80	0.17	−3.62 to 17,23	0.17

Myostatin concentration was the outcome variable and BCS, myostatin and ActRIIB gene and protein expression data for the individual skeletal muscles was offered as explanatory variables.

## Discussion

This study presents preliminary data which provide the first indication of a possible association between BCS and myostatin gene expression and secretion in horses and ponies.

The increased gene expression of myostatin in skeletal muscles of obese animals is in agreement with similar data for mice where myostatin mRNA levels were significantly greater in *tibialis anterior* muscle in *ob/ob* mice compared to wild-type mice [20]. In that study [20], the expression of ActRIIB was not different between lean and obese animals for skeletal muscle, whereas in the current study, ActRIIB mRNA was significantly down-regulated in three out of the four skeletal muscles studied. This may be suggestive of some element of negative feedback regulation between myostatin and ActRIIB.

Increased expression of myostatin protein has been identified in the *vastus lateralis* muscle from extremely obese human subjects (BMI ≥40 kg/m^2^) [21]. Perhaps the lack of statistical significance observed in the current study may be due to absolute differences in body fat content between species. Obese horses and ponies were found to have up to 30% body fat recorded in a previous study [32], which is considerably lower than the body fat content of morbidly obese humans which was found to average 48.5% [33]. The finding of altered myostatin and ActRIIB mRNA expression in muscle without parallel changes in protein expression has previously been shown [34], [35]. It is known that the mRNA expression of a particular gene is not always predictive of protein expression, and the correlation between the two can vary significantly [36]. There are several possible explanations for the differences between the gene and protein expression including variation in protein half-lives, complex post-transcriptional mechanisms, and different sensitivities in methodologies for detecting mRNA and protein expressions [37].

Circulating concentrations of myostatin were significantly higher in obese than in lean animals in the current study. This is consistent with previous observations of increased myostatin secretion from myotubes derived from muscle of extremely obese humans [21]. In the current study there was one clear outlier in our lean group of animals for both circulating concentrations and mRNA expression of myostatin which upon investigation was found to be the Welsh pony mare (Horse 2; Table 1). It could be speculated that this may be indicative of an increased propensity towards obesity based on a finding from a murine study in which obesity-susceptible strain of mice (C57BL/6) had increased mRNA expression of myostatin in skeletal muscle compared to an obesity-resistant strain of mice, SWR/J [38].

Myostatin gene expression was generally low in adipose tissues but was significantly higher in crest fat from obese than lean animals. Increased fat deposition in this subcutaneous fat depot along the nuchal crest of the neck in horses and ponies has been associated with laminitis risk [39], hyperinsulinemia [40], and has been proposed to be an important source of proinflammatory cytokines [41]. Differences in myostatin gene expression between crest fat samples from lean and obese animals were not reflected in protein expression in this tissue. Data indicated that neither myostatin precursor nor ActRIIB proteins were detectable in the crest fat of either lean or obese animals by the methods used in the current study. This is in agreement with data presented in an earlier study which similarly failed to detect either myostatin precursor or ActRIIB proteins in crest and other adipose tissues from lean (BCS<4/9) animals [13].

These preliminary data offer some evidence that the ‘myostatin system’ may differ at both the gene and protein level in lean and obese horses and ponies. Further work is needed, and these findings now provide the basis for future prospective studies in horses and ponies to explore the previous speculation from human studies [21] that circulating myostatin levels and/or associated factors might act as biological marker(s) for metabolic conditions including obesity.

## References

[1] Giles SL, Rands SA, Nicol CJ, Harris PA (2014) Obesity prevalence and associated risk factors in outdoor living domestic horses and ponies. PeerJ 2: e299. 24711963 10.7717/peerj.299 PMC3970797

[2] Harker IJ, Harris PA, Barfoot CF (2011) The body condition score of leisure horses competing at an unaffiliated championship in the UK. Journal of Equine Veterinary Science 31: 253–254.

[3] Owers R, Chubbock S (2013) Fight the fat! Equine veterinary journal. 45: 5–5. 10.1111/evj.12008 23231382

[4] Dugdale AHA, Curtis GC, Harris PA, Argo CM (2011) Assessment of body fat in the pony: Part I. Relationships between the anatomical distribution of adipose tissue, body composition and body condition. Equine veterinary journal 43: 552–561. 21496091 10.1111/j.2042-3306.2010.00330.x

[5] Webb AI, Weaver BMQ (1979) Body Composition of the Horse. Equine veterinary journal 11: 39–47. 428363 10.1111/j.2042-3306.1979.tb01295.x

[6] McPherron A, Lawler A, Lee S (1997) Regulation of skeletal muscle mass in mice by a new TGF-beta superfamily member. Nature 387: 83–90. 9139826 10.1038/387083a0

[7] Joulia D, Bernardi H, Garandel W, Rabenoelina F, Vernus B, et al (2003) Mechanisms involved in the inhibition of myoblast proliferation and differentiation by myostatin. Experimental Cell Research 286: 263–275. 12749855 10.1016/s0014-4827(03)00074-0

[8] Langley B, Thomas M, Bishop A, Sharma M, Gilmour S, et al (2002) Myostatin Inhibits Myoblast Differentiation by Down-regulating MyoD Expression. Journal of Biological Chemistry 277: 49831–49840. 12244043 10.1074/jbc.M204291200

[9] Whittemore L-A, Song K, Li X, Aghajanian J, Davies MV, et al (2003) Inhibition of myostatin in adult mice increases skeletal muscle mass and strength. Biochemical and Biophysical Research Communications 300: 965–971. 12559968 10.1016/s0006-291x(02)02953-4

[10] Benny Klimek ME, Aydogdu T, Link MJ, Pons M, Koniaris LG, et al (2010) Acute inhibition of myostatin-family proteins preserves skeletal muscle in mouse models of cancer cachexia. Biochemical and Biophysical Research Communications 391: 1548–1554. 20036643 10.1016/j.bbrc.2009.12.123

[11] Wagner KR, Fleckenstein JL, Amato AA, Barohn RJ, Bushby K, et al (2008) A phase I/IItrial of MYO-029 in adult subjects with muscular dystrophy. Annals of Neurology 63: 561–571. 18335515 10.1002/ana.21338

[12] Hill JJ, Davies MV, Pearson AA, Wang JH, Hewick RM, et al (2002) The Myostatin Propeptide and the Follistatin-related Gene Are Inhibitory Binding Proteins of Myostatin in Normal Serum. Journal of Biological Chemistry 277: 40735–40741. 12194980 10.1074/jbc.M206379200

[13] Morrison PK, Bing C, Harris PA, Maltin CA, Grove-White D, et al (2014) Post-Mortem Stability of RNA in Skeletal Muscle and Adipose Tissue and the Tissue-Specific Expression of Myostatin, Perilipin and Associated Factors in the Horse. PLoS ONE 9: e100810. 24956155 10.1371/journal.pone.0100810 PMC4067385

[14] Dilger AC, Spurlock ME, Grant AL, Gerrard DE (2010) Myostatin null mice respond differently to dietary-induced and genetic obesity. Animal Science Journal = Nihon Chikusan Gakkaihō 81: 586–593. 20887312 10.1111/j.1740-0929.2010.00776.x

[15] Hamrick MW, Pennington C, Webb CN, Isales CM (2006) Resistance to body fat gain in ‘double-muscled’ mice fed a high-fat diet. International Journal of Obesity 30: 868–870. 16404405 10.1038/sj.ijo.0803200

[16] Guo W, Flanagan J, Jasuja R, Kirkland J, Jiang L, et al (2008) The effects of myostatin on adipogenic differentiation of human bone marrow-derived mesenchymal stem cells are mediated through cross-communication between Smad3 and Wnt/beta-catenin signaling pathways. The Journal Of Biological Chemistry 283: 9136–9145. 18203713 10.1074/jbc.M708968200 PMC2431017

[17] Hirai S, Matsumoto H, Hino N, Kawachi H, Matsui T, et al (2007) Myostatin inhibits differentiation of bovine preadipocyte. Domestic Animal Endocrinology 32: 1–14. 16431073 10.1016/j.domaniend.2005.12.001

[18] Fournier B, Murray B, Gutzwiller S, Marcaletti S, Marcellin D, et al (2012) Blockade of the Activin Receptor IIB Activates Functional Brown Adipogenesis and Thermogenesis by Inducing Mitochondrial Oxidative Metabolism. Molecular and Cellular Biology 32: 2871–2879. 22586266 10.1128/MCB.06575-11 PMC3416189

[19] Shan T, Liang X, Bi P, Kuang S (2013) Myostatin knockout drives browning of white adipose tissue through activating the AMPK-PGC1α-Fndc5 pathway in muscle. The FASEB Journal 27: 1981–1989. 23362117 10.1096/fj.12-225755 PMC3633817

[20] Allen DL, Cleary AS, Speaker KJ, Lindsay SF, Uyenishi J, et al (2008) Myostatin, activin receptor IIb, and follistatin-like-3 gene expression are altered in adipose tissue and skeletal muscle of obese mice. American Journal Of Physiology Endocrinology And Metabolism 294: E918–E927. 18334608 10.1152/ajpendo.00798.2007

[21] Hittel DS, Berggren JR, Shearer J, Boyle K, Houmard JA (2009) Increased secretion and expression of myostatin in skeletal muscle from extremely obese women. Diabetes 58: 30–38. 18835929 10.2337/db08-0943 PMC2606890

[22] Cleasby ME, Jarmin S, Eilers W, Elashry M, Andersen DK, et al. (2014) Local overexpression of the myostatin propeptide increases glucose transporter expression and enhances skeletal muscle glucose disposal. E814–E823 p. 10.1152/ajpendo.00586.2013 PMC3962614 24473441

[23] Dall'Olio S, Fontanesi L, Nanni Costa L, Tassinari M, Minieri L, et al. (2010) Analysis of Horse Myostatin Gene and Identification of Single Nucleotide Polymorphisms in Breeds of Different Morphological Types. Journal of Biomedicine and Biotechnology 2010. 10.1155/2010/542945 PMC2913906 20706663

[24] Hill EW, McGivney BA, Gu J, Whiston R, Machugh DE (2010) A genome-wide SNP-association study confirms a sequence variant (g.66493737C>T) in the equine myostatin (MSTN) gene as the most powerful predictor of optimum racing distance for Thoroughbred racehorses. BMC Genomics 11: 552–552. 20932346 10.1186/1471-2164-11-552 PMC3091701

[25] Petersen JL, Mickelson JR, Rendahl AK, Valberg SJ, Andersson LS, et al (2013) Genome-Wide Analysis Reveals Selection for Important Traits in Domestic Horse Breeds. PLoS Genet 9: e1003211. 23349635 10.1371/journal.pgen.1003211 PMC3547851

[26] Kohnke J (1992) Feeding and Nutrition: The making of a champion: Birubi Pacific, Pymble. 163–166 p.

[27] Ahn K, Bae J-H, Nam K-H, Lee C-E, Park K-D, et al (2011) Identification of reference genes for normalization of gene expression in thoroughbred and Jeju native horse(Jeju pony) tissues. Genes & Genomics 33: 245–250.

[28] Bogaert L, Van Poucke M, De Baere C, Peelman L, Gasthuys F, et al (2006) Selection of a set of reliable reference genes for quantitative real-time PCR in normal equine skin and in equine sarcoids. BMC Biotechnology 6: 24. 16643647 10.1186/1472-6750-6-24 PMC1484482

[29] Schmittgen TD, Livak KJ (2008) Analyzing real-time PCR data by the comparative CT method. Nat Protocols 3: 1101–1108. 18546601 10.1038/nprot.2008.73

[30] Smith PK, Krohn RI, Hermanson GT, Mallia AK, Gartner FH, et al (1985) Measurement of protein using bicinchoninic acid. Analytical Biochemistry 150: 76–85. 3843705 10.1016/0003-2697(85)90442-7

[31] Dugdale AHA, Grove-White D, Curtis GC, Harris PA, Argo CM (2012) Body condition scoring as a predictor of body fat in horses and ponies. The Veterinary Journal. 10.1016/j.tvjl.2012.03.024 22578691

[32] Argo CM, Curtis GC, Grove-White D, Dugdale AHA, Barfoot CF, et al. (2012) Weight loss resistance: A further consideration for the nutritional management of obese Equidae. The Veterinary Journal. 10.1016/j.tvjl.2012.09.020 23117030

[33] Vijgen GHEJ, Bouvy ND, Teule GJJ, Brans B, Schrauwen P, et al (2011) Brown Adipose Tissue in Morbidly Obese Subjects. PLoS ONE 6: e17247. 21390318 10.1371/journal.pone.0017247 PMC3044745

[34] Baumann AP IC, Grasser WA, Paraklar VM (2003) Myostatin expression in age and denervation-induced skeletal muscle atrophy. J Musculoskel Neuron Interact 3: 8–16. 15758361

[35] Smith IJ, Aversa Z, Alamdari N, Petkova V, Hasselgren P-O (2010) Sepsis downregulates myostatin mRNA levels without altering myostatin protein levels in skeletal muscle. Journal of Cellular Biochemistry 111: 1059–1073. 20677217 10.1002/jcb.22796

[36] Guo Y, Xiao P, Lei S, Deng F, Xiao GG, et al (2008) How is mRNA expression predictive for protein expression? A correlation study on human circulating monocytes. Acta Biochimica et Biophysica Sinica 40: 426–436. 18465028 10.1111/j.1745-7270.2008.00418.x

[37] Greenbaum D, Colangelo C, Williams K, Gerstein M (2003) Comparing protein abundance and mRNA expression levels on a genomic scale. Genome Biology 4: 117. 12952525 10.1186/gb-2003-4-9-117 PMC193646

[38] Lyons J-A, Haring JS, Biga PR (2010) Myostatin Expression, Lymphocyte Population, and Potential Cytokine Production Correlate with Predisposition to High-Fat Diet Induced Obesity in Mice. PLoS ONE 5: e12928. 20877574 10.1371/journal.pone.0012928 PMC2943928

[39] Carter RA, Treiber KH, Geor RJ, Douglass L, Harris PA (2009) Prediction of incipient pasture-associated laminitis from hyperinsulinaemia, hyperleptinaemia and generalised and localised obesity in a cohort of ponies. Equine veterinary journal 41: 171–178. 19418747 10.2746/042516408x342975

[40] Carter RA, Geor RJ, Burton Staniar W, Cubitt TA, Harris PA (2009) Apparent adiposity assessed by standardised scoring systems and morphometric measurements in horses and ponies. The Veterinary Journal 179: 204–210. 18440844 10.1016/j.tvjl.2008.02.029

[41] Bruynsteen L, Erkens T, Peelman L, Ducatelle R, Janssens G, et al (2013) Expression of inflammation-related genes is associated with adipose tissue location in horses. BMC Veterinary Research 9: 1–9. 24295090 10.1186/1746-6148-9-240 PMC4220830

